# Correction: Padlocking dihydrofurannulation for the control of small degree of helicity built on a fused-tetracyclic core

**DOI:** 10.1039/d4sc90186j

**Published:** 2024-09-25

**Authors:** Arthur Gaucherand, Expédite Yen-Pon, Diego García-López, Jean-Valère Naubron, Sara Chentouf, Michel Giorgi, Stéphane Humbel, Marion Jean, Jean Rodriguez, Damien Bonne

**Affiliations:** a Aix Marseille Université, CNRS, Centrale Marseille, iSm2 Marseille France; b Aix Marseille Université, CNRS, Centrale Marseille, FSCM, Spectropole Marseille France

## Abstract

Correction for ‘Padlocking dihydrofurannulation for the control of small degree of helicity built on a fused-tetracyclic core’ by Arthur Gaucherand *et al.*, *Chem. Sci.*, 2024, **15**, 7300–7307, https://doi.org/10.1039/D4SC00745J.

The authors regret there was missing information in the Acknowledgements section of the original article. The corrected Acknowledgements section for this article is shown below.

Financial support from the Agence Nationale pour la Recherche (ANR-21-CE07-0036), Aix-Marseille Université, the Centre National de la Recherche Scientifique (CNRS), and Centrale Marseille is gratefully acknowledged. We warmly thank Cecilia Sasso D'Elia for the synthesis of compound **1** during her PhD in our group. This work was supported by the computing facilities of the CRCMM, ‘Centre Régional de Compétences en Modélisation Moléculaire de Marseille’.

The Royal Society of Chemistry apologises for these errors and any consequent inconvenience to authors and readers.

